# The Value of Reversible Carbon Storage in a Zero-Emissions World

**DOI:** 10.1021/acs.est.6c00333

**Published:** 2026-06-07

**Authors:** Allegra C. Mayer, Jerome Dumortier, Zeke Hausfather, Jennifer Pett-Ridge, Eric W. Slessarev

**Affiliations:** † 4578 Lawrence Livermore National Laboratory, Physical Life Sciences Division, Livermore, California 94551, United States; ‡ 10668 Indiana University Indianapolis, O’Neill School of Public and Environmental Affairs, Indianapolis, Indiana 46202, United States; § 524125 Stripe Inc., South San Francisco, California 94080, United States; ∥ Berkeley Earth, Berkeley, California 94705, United States; ⊥ Life and Environmental Sciences Department, University of California, Merced, California 95343, United States; # Innovative Genomics Institute, University of California, Berkeley, California 94704, United States; ∇ Yale University, Department of Ecology and Evolutionary Biology, New Haven, Connecticut 06511, United States; ○ Yale University, Yale Center for Natural Carbon Capture, New Haven, Connecticut 06511, United States

**Keywords:** carbon dioxide removal, reversible carbon storage, durable carbon storage, durability, warming reduction, economics, cover crops

## Abstract

Atmospheric carbon dioxide removal (CDR) is required to stabilize global temperature. CDR can be achieved via ecosystem-based approaches that are cost-effective but reversible (e.g., soil and forest management) or by more durable but expensive approaches (e.g., direct air capture coupled with geologic storage). Here, we examine trade-offs between these approaches, focusing on timing, climate impacts, and cost. We simulated reversible carbon accrual for a range of CDR contract structures using a general minimalist model of ecosystem carbon cycling, and parameterized it to simulate US agricultural soil management—specifically cover cropping—as a case study. We then quantified the resulting impact on atmospheric carbon and global temperature using a climate model emulator. We find that maintaining a patchwork of reversible CDR projects by replacing lapsed projects with new projects can reduce warming by 22–195 μ°C in 2100 and that the magnitude of this cooling effect depends on how effectively the patchwork is maintained. Long-term maintenance of reversible CDR projects requires institutional stability that cannot be guaranteed over multiple decades. Consequently, effective CDR ultimately requires replacing reversible projects with durable projects. To address this problem, we modeled the cost of replacing reversible agricultural soil CDR with geologic CDR. We found that using reversible CDR as a bridge to durable CDR is potentially more cost-effective as a global cooling strategy (0.20–0.81 billion USD per μ°C avoided) than perpetual maintenance of reversible CDR (0.32–1.31 billion USD per μ°C avoided) or an immediate transition to durable CDR (1.37–2.19 billion USD per μ°C avoided). However, we emphasize that institutional commitments to maintain reversible CDR projects cannot be guaranteed. Reliance on reversible CDR as a bridge to durable CDR therefore carries an unknown amount of risk and will only function if efforts to maintain reversible CDR are robust.

## Graphical abstract

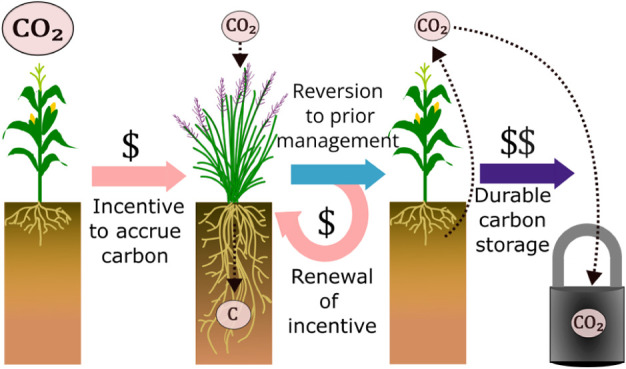

## Introduction

Carbon dioxide removal (CDR) must complement decarbonization to meet climate mitigation targets.^1^ CDR pathways that yield potentially durable storage are currently expensive, energetically demanding, or not yet scalable. These pathways include biomass with carbon removal and storage (BiCRS), direct air capture with geologic storage (DACS), enhanced rock weathering (ERW), and ocean alkalinity enhancement (OAE). By contrast, ecosystem-based CDR pathways, such as organic carbon sequestration in forests and soils, provide many environmental benefits and are inexpensive, low-energy, and scalable. However, these pathways are not durable because plant biomass and soil organic matter can burn or decompose, returning to the atmosphere.^2–4^ It remains unclear how reversible ecosystem-based CDR should be integrated with more durable CDR pathways to mitigate climate change.

The effectiveness of CDR depends on the duration and timing of CO_2_ emissions. Reversible CDR will be most effective if it is initiated early and maintained until after peak warming.^5^ If storage is reversed prior to climate stabilization, the resulting pulse of CO_2_ could create a sharper climate forcing response and higher near-term temperatures than a case with a smoother atmospheric CO_2_ trajectory without any reversible storage.^5^ Carbon that is re-emitted from ecosystems prior to reaching peak warming has virtually no long-term climate mitigation effect when compared to that of durable CDR.^6,7^ The effectiveness of ecosystem-based CDR also interacts with future emissions trajectories: reversible storage can reduce peak warming when combined with strong fossil fuel emissions reductions or delay peak warming when combined with medium emissions reductions.^6,8^ These facts imply that the social and economic factors governing the implementation rate and maintenance of land management-based CDR will determine its contribution to warming reduction.

Ecosystem-based CDR projects are typically commissioned by governments or private companies but are implemented by individual landowners, which distributes the risk of CDR reversal across space and time. Institutions incentivize CDR by establishing a multitude of short-term contracts that compensate landowners for adopting carbon-sequestering management practices, replacing lapsed contracts over time. This distributed approach to CDR is known as “horizontal stacking.” ^9,10^ A patchwork of CDR contracts across space and time can achieve the same effects as one large-scale project,^11^ provided that the institution managing the patchwork upholds its commitments. Recent carbon accounting guidance recommends implementing horizontal stacking by renewing short-term commitments, together with contributions to reserve or buffer pools to account for reversal risks.^12^ Critically, this strategy rests on the assumption that the release of ecosystem carbon occurs at relatively small scales. Replacing carbon storage may become impossible if climate change or large-scale economic shocks reduce the overall capacity of ecosystems to store carbon.

While horizontal stacking of reversible carbon storage can affect the trajectory of climate change, a more secure approach is to prioritize durable storage pathways that have a lower probability of reversal. A range of CDR approaches, including DACS, BiCRS, ERW, and OAE, store CO_2_ in putatively durable geologic reservoirs with millennial residence times or greater.^13^ Durable CDR is relatively nascent today;^13^ however, the sector is rapidly expanding and is likely to reach a scale of tens of millions of tons per year by 2030.^14^ Current costs of durable CDR generally exceed $200 per metric ton of CO_2_-equivalent (Mg^–1^ CO_2_-e), with some pathways (e.g., DACS) above $500 Mg^–1^ CO_2_-e.^14,15^ Although these costs are expected to decrease over time due to learning through larger-scale deployments, they will likely remain over $100 Mg^–1^ CO_2_-e for the next 50 years.^14,16^ Scaling depends on investments, regulatory constraints for permitting, technological development, and cultural, social, and environmental justice constraints around projects in vulnerable areas. By contrast, reversible CDR through ecosystem pathways can be delivered at much lower costs ($0–100) though with a lower maximum annual rate of removal (estimates range from 8–80 million tons CO_2_ per year by 2050). From an investment perspective, we frame durable CDR and storage as “buying” carbon storage as an expensive but one-time investment, in contrast to “renting” reversible storage via horizontal stacking, which is less expensive but must be maintained.^10,17^

Comparing reversible and durable carbon storage is fraught because the effect of reversible carbon storage on the climate depends on the storage duration, which is uncertain. Ton-year accounting and the like-for-like principle have been proposed as solutions for valuing shorter-duration carbon storage.^18–22^ However, these frameworks do not quantitatively account for the risks and benefits of competition between end members of reversible carbon storage and durable storage pathways over time.^23,24^ Our analysis closes this knowledge gap by evaluating (1) the climate forcing associated with horizontal stacking of ecosystem-based carbon storage (using agricultural management such as cover cropping, deep-rooting crops, and perennialization as a case study) and (2) cost tradeoffs among renting agricultural management-based storage, buying durable storage, or blending a combination of the two strategies. To compare these scenarios, we integrated three models representing the dynamics of carbon storage across a patchwork of CDR projects, global temperature effects of the patchwork, and associated costs. We then analyzed a range of trajectories for transitioning from renting reversible CDR storage projects to buying durable geologic-scale carbon storage.

We used this modeling framework to explore two main hypotheses. First, we assess the climate and cost tradeoffs associated with implementing new reversible CDR projects (with faster accrual rates) versus maintaining CDR projects for longer (delaying release). We hypothesize that maximizing negative radiative forcing per dollar spent over a 50- to 100-year period requires the maintenance of CDR projects that are no longer accruing carbon, but that the maintenance of some CDR projects can lapse without increasing warming, provided that lost CDR is replaced with new CDR. Second, we quantify the cost of continuously renting reversible projects until eventually buying durable storage. We hypothesize that as the cost of durable CDR declines, the proportion of carbon stored in durable CDR projects should be able to financially compete with reversible projects in the long term, suggesting the use of reversible projects as a bridging mechanism rather than a final storage solution.

## Methods

We represent reversible CDR using a generalized 1-pool model of ecosystem carbon storage that can simulate any system affected by input (flow from the atmosphere to the system), decay (intrinsic rerelease of carbon), and the duration of the CDR practice. For the purposes of this proof-of-concept analysis, we consider a single ecosystem-based CDR pathway: cover cropping. Cover cropping is an agricultural practice that aims to enhance soil organic carbon storage by increasing photosynthetic inputs during fallow periods. We chose cover cropping because it is a well-understood practice but not widely practiced year-on-year in the USA,^25^ and hence adoption is likely to be additional. Cover cropping also does not directly compete with commodity crop production or require the import of exogenous biomass.^16^ To represent permanent storage, we focused on DACS. We use DACS as a proxy for durable CDR pathways as it has a large potential and known current costs,^26^ although other approaches, including hybrid ecosystem-technological approaches, will likely represent a larger share of near-term permanent CDR deployment.^27^ We acknowledge that durable geologic storage via BiCRS or DACS is not necessarily permanent because there is a nonzero potential for leaks.^28^ Furthermore, there are ecosystem-based approaches that fit in an intermediate storage durability category^7^ (e.g., surface-applied biochar), complicated by competition for biomass between approaches. We imagine a simplified scenario in which there is no competition between managed ecosystem and geologic storage pathways. We focus on short-term approaches that depend on the maintenance of land management solutions rather than hybrid ecosystem-technological solutions.

First, we implemented a minimalist biogeochemical model to track annual carbon sequestration and release in response to the ecosystem CDR practice. We parameterized the model to simulate the effects of cover cropping, a case-study soil management practice that is thought to yield CDR. Next, we combined the biogeochemical model with a land-use model to track carbon sequestration and release across a patchwork of CDR projects, starting in 2025. We simulated different contract renewal rates for the patchwork; these rates represent the overall efficacy of the patchwork in maintaining carbon storage by replacing lapsed projects. Because we assume perfect foresight, a carbon buffer pool was not included in our analysis. Depending on the scenario, not all carbon released was added to the atmosphere; instead, a fraction of released carbon could be stored in durable CDR projects based on the expansion of the DACS technology. We fed the carbon fluxes from the land-use model into the finite amplitude impulse response (FaIR 1.6.4) model to calculate the associated effects on global temperature.^29–31^ Lastly, we used an economic model to attribute costs to the various CDR pathways and temperature effects.

### Soil Organic Carbon Model

We simulated soil carbon storage due to cover cropping with a linear one-pool soil organic carbon (SOC) model. The effect of cover crops on SOC remains debated,^32–35^ but for the purposes of this analysis, we assumed that cover crops increase SOC stocks. We define the management practice as either implementing cover crops or managing land with the business-as-usual practice (i.e., no cover crops). The rate of change in SOC was governed by a single differential equation:^36^

1 $$dC_{soil}/dt=nI-kC_{soil}$$

where *C_soil_* is the soil carbon stock (Mg C ha^–1^), *I* is the rate of carbon input (Mg C ha^–1^ y^–1^), *n* is the fraction of carbon input that enters the soil (unitless), and *k* is a first-order decay constant (y^–1^). SOC stocks are calculated on an annual timestep using the integral of eq 1):

2 $$C_{soil}\left(t\right)=\frac{n}{k}I-\left(\frac{n}{k}I-C_{0}\right)e^{-k(t-t_{0})}$$

where *C*_0_ is the SOC stock (Mg C ha^–1^) at time *t*_0_. We parameterized this equation to represent mean estimates of soil carbon accrual from implementing cover crops on annual croplands in the temperate US^27,47–51^ (see detailed discussion of parameters in the Supporting Information). Our modeled soil carbon accrual rate is conservative but well within the range of values across the US for the US Department of Agriculture technical guidelines for accounting for cover cropping.^37^

To capture the time dynamics of SOC accrual, we solved a piecewise equation that tracked (1) initial SOC under business-as-usual management, (2) accrual of SOC following the implementation of cover cropping for the duration of the cover-cropping contract, and (3) carbon loss after the termination of the contract and cessation of cover cropping. We calculated net carbon storage by subtracting business-as-usual carbon stock from the solution at each time and location (see equations in the Supporting Information). This model accounted for the variable rate of soil carbon accrual over time: a patch that was contracted three times in a row accrued at a slower rate than a patch of land contracted for the first time (Figure 1). We note that these parameters could be adjusted for any ecological storage pathway that approaches equilibrium, in which case carbon accumulation dynamics would vary depending on the rate of carbon input and decay.

**Figure 1:**
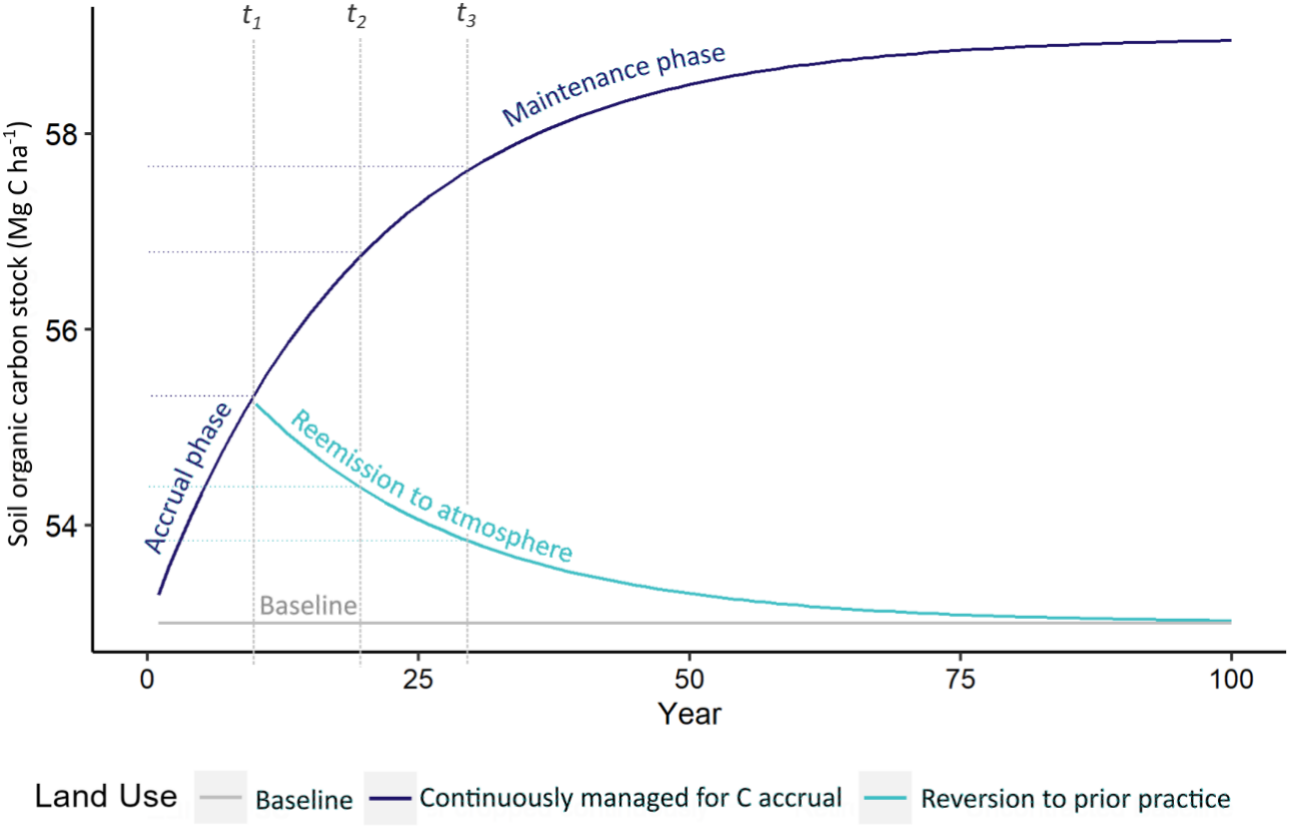
Simulated soil organic carbon stock over time using a one-pool model, given (1) a steady-state baseline (gray line) of 53 Mg C ha^–1^, (2) land under continuous CDR management practices such as cover cropping (purple line), and (3) land that had been under a cover crop regime for 10 years but returned to the original management practice with baseline inputs (blue line). Soil CDR contracts that are renewed through time *t_2_*, *t_3_*, or later have diminishing carbon accrual returns over time, and contracted projects reach a maintenance phase of carbon accrual rather than the accrual phase. Soil carbon release from land reverted to previous practices (blue line) follows the same assumptions for decomposition as in the other two cases, but with initial carbon as the value of the soil carbon stock whenever the contract ended, and carbon inputs shifting back to the original management practice. Importantly, although soil carbon is being released after reversion to prior practice, total carbon stock remains higher than the baseline for decades following reversion under these conditions.

### Patchwork Land-Use Model

We developed a land-use model to track carbon accrual and loss as patches of land shift into and out of reversible carbon storage over time. Each patch represented an aggregated area of land with a shared management history, meaning that cover crops were implemented and lapsed in the same years for all land within the patch. To construct the patchwork, we simulated a range of possible management contract outcomes with renewal rates of 0%, 25%, 50%, 75%, and 100%, as well as contract lengths of 5–20 years in increments of 5 years. For the main analysis, we report results from 10-year contracts and include results from all contract lengths in the Supporting Information. We used a total land area of 91 million hectares, which is equivalent to the area planted in corn, soybeans, or wheat in the USA in 2022,^38^ and we limited total land under soil carbon accrual practices (A_t_) to 25% (i.e., 22.75 million ha) at any point in time, which is slightly higher than current enrollment rates for incentivized cropland conservation programs in the USA (e.g., Environmental Quality Incentive Program).^39^ Land that reverted to the baseline practice at the end of the contract was replaced with the same amount of land being enrolled in the carbon-removal practice until all land had been enrolled at least once. Total carbon removal was calculated by summing carbon stored across all patches over time. Patchwork model equations are found in Supporting Information.

### Transition from Reversible to Durable CDR

In the patchwork land-use model, carbon was gradually released back into the atmosphere when contracts were not renewed or replaced. We modeled scenarios in which net carbon released was durably removed through DACS at the prevailing cost in the year of release. Since there is uncertainty about how quickly DACS will scale over time, we evaluated reversible (“rent”) to durable (“buy”) transition pathways to understand the degree to which reversible carbon removal and storage might bridge a gap to durable carbon storage. We assumed that the fraction of released soil carbon that would be removed via DACS grew as a logistic curve, approaching 100% removal of carbon emitted by either 2050 or 2075, which we labeled Early DACS Transition and Later DACS Transition, respectively. In all cases with durable storage, net carbon removal occurred by the end of the simulation, as all carbon released from soil after 2075 at the latest was durably removed through DACS. To compare the effects both in terms of temperature and cost, we also simulated “rent-only” and “buy-only” scenarios. The “rent-only” scenarios assumed no durable storage, or no cost-competitive durable storage, and the “buy-only” scenarios assumed immediate durable storage via DACS, with a net CDR trajectory that replicates the carbon removal achieved from the different ecological CDRs based on renewal rate. We note that with the development of new CDR technologies, more CDR will be available by 2050 than what is modeled in this study.^16,40,41^ The DACS diffusion curves used in this analysis are available in the Supporting Information (Figure S1).

### Translation to Climate Warming Reduction

We translated spatially cumulative carbon fluxes from the patchwork land-use model to climate forcing effects using a climate model emulator, the *FaIR model*.^29,30^ Specifically, we added rates of soil carbon uptake or emission (in units of Mg C y^–1^) to global greenhouse gas emissions scenarios for two Shared Socioeconomic Pathways (SSP) and Representative Concentration Pathways (RCP): (1) SSP1-2.6, a scenario with global cooperation and major emissions reductions, and (2) SSP 2–4.5, which simulates moderate global emissions reductions.^42^ We present results using a baseline of SSP1-2.6, as the major shift to durable storage simulated in this research is most consistent with this land-use and emissions storyline. The FaIR model^29^ calculates the global impact of carbon fluxes on radiative forcing and mean global temperature. For each contract renewal rate, the change in carbon fluxes and associated change in climate forcing were compared to climate forcing outcomes assuming only the baseline emissions (e.g., SSP1-2.6), and we report the relative change in warming. Results for a baseline of SSP2-4.5 and for contract lengths from 5 to 20 years are available in the Supporting Information (Figures S5 and S6).

### Economic Aspects

There are three cost components associated with the patchwork land-use model: the costs of reversible storage, the cost of durable storage, and the discount rate. Regarding the cost of reversible soil storage, we assumed a practice-based, as opposed to a results-based, payment policy. That is, landowners are compensated per hectare of land enrolled in the practice. A results-based policy would compensate landowners based on the carbon removed on an annual basis, but since the removal rate decreases over time (Figure 1), payments received would approach zero in the long run, making it unlikely for a landowner to renew a contract. The disadvantage of practice-based payments is that landowners get compensated for the practice (even in the very long run), even if no carbon is accumulated in the soil. We discuss the implications of this choice further in the Supporting Information.

We considered three carbon payment paths, which we labeled low C value, medium C value, and high C value (Figure S3). The growth rate for each path was set to 1%, and the starting values in 2025 were $50, $125, and $200 per metric ton of CO_2_ for low, medium, and high price paths, respectively. For the three price paths, we assumed that the maximum values were $250, $500, and $1000 (real 2025 dollars) per metric ton of CO_2_ for the low, medium, and high values, respectively. If contracts were renewed, the payment started with the value prevailing in the renewal year. The price was measured in USD per hectare of land enrolled in cover cropping. As with other components of our analysis, there is substantial uncertainty regarding the payment to landowners for reversible carbon removal and storage, which is reflected in the carbon value paths. The resulting carbon price trajectories are in line with published values.^43^

The future cost of verifiable durable CDR is highly uncertain, with current cost estimates for DACS (a proxy for durable CDR) ranging from $600–$1000 Mg^–1^ CO_2_-e.^44^ A similar issue arises when projecting the cost in the long run. In this analysis, we assumed three DACS cost trajectories consistent with estimates starting at $500, $750, and $1000 Mg^–1^ CO_2_-e in 2025 and declining to $150, $250, and $500 Mg^–1^ CO_2_-e in the long term (Figure S1b). These values should be comparable to the carbon payments per hectare, which are increasing over time. We also include the rent-only scenario without any durable storage, under which DACS or other durable CDR pathways never become cost-competitive.

Note that, unlike in Herzog et al. (2003), we allow the possibility that the carbon price grows at a different rate than the discount rate. We analyzed discount rates ranging from 0% to 5% in steps of 2.5 percentage points (Figure S4). We focus our discussion of the economic cost on the central scenarios in terms of discount rate (2.5%), carbon payments per hectare (Medium C Value), and DAC cost (Medium DAC Cost). In this analysis, costs are represented as net present value in the years 2075, 2100, 2200, 2300, and 2500. Higher discount rates resulted in a lower net present value of carbon removal because high future costs are discounted at a higher rate. Higher (private) discount rates emphasize near-term outcomes and make reversible storage more attractive, while lower (social) discount rates place greater weight on long-term climate damages that grow with population, assets, and economic activity. Our analysis models temperature impacts over a multidecadal and century horizon, adopting a long-term societal perspective, which justifies our use of the lower end of the discount-rate range. We note that the discount rate in our model affects only the cost calculations and does not alter the physical trajectory of reversible versus durable storage.

## Results

Here, we focus on the (1) carbon fluxes to and from the atmosphere, (2) temperature anomalies, and (3) economic costs of the pathways. We focus our discussion on central scenarios, with additional results in the Supporting Information.

### Carbon Fluxes

First, we focus on “rent-only” scenarios, in which cover cropping was used to contribute to CDR in the absence of durable storage. In these scenarios, carbon accrued following cover cropping but was re-emitted after the cover-cropping contract lapsed. The dynamics of re-emission were relatively slow: carbon storage remained greater than the baseline for more than 20 years after cover cropping ended (Figure 1). Re-emission dynamics depend on the structure and parameterization of the biogeochemical model and might happen faster (or slower) in reality.

Assuming 100% renewal of cover cropping contracts, we found that enrolling the maximum land area of 22.75 million hectares and renewing over the entire period removed a total of 500 Mg CO_2_-e (136.3 Mg C). At renewal rates less than 100%, the land available for new cover cropping contracts was eventually exhausted, and a fraction of the land being cover cropped reverted to conventional management, releasing carbon back to the atmosphere (Figure 2A). Maintaining enrollment of land in cover cropping longer delayed the exhaustion of available land and the subsequent release of carbon back into the atmosphere beyond 2250 (Figure 2B). Lower renewal rates (25%) led to earlier carbon releases of 411 Mg CO_2_-e (112 Mg C) in 2125 out of the maximum 458 Mg CO_2_-e (125 Mg C) accrued, with less than 1% of the accrued carbon remaining after 2175.

**Figure 2:**
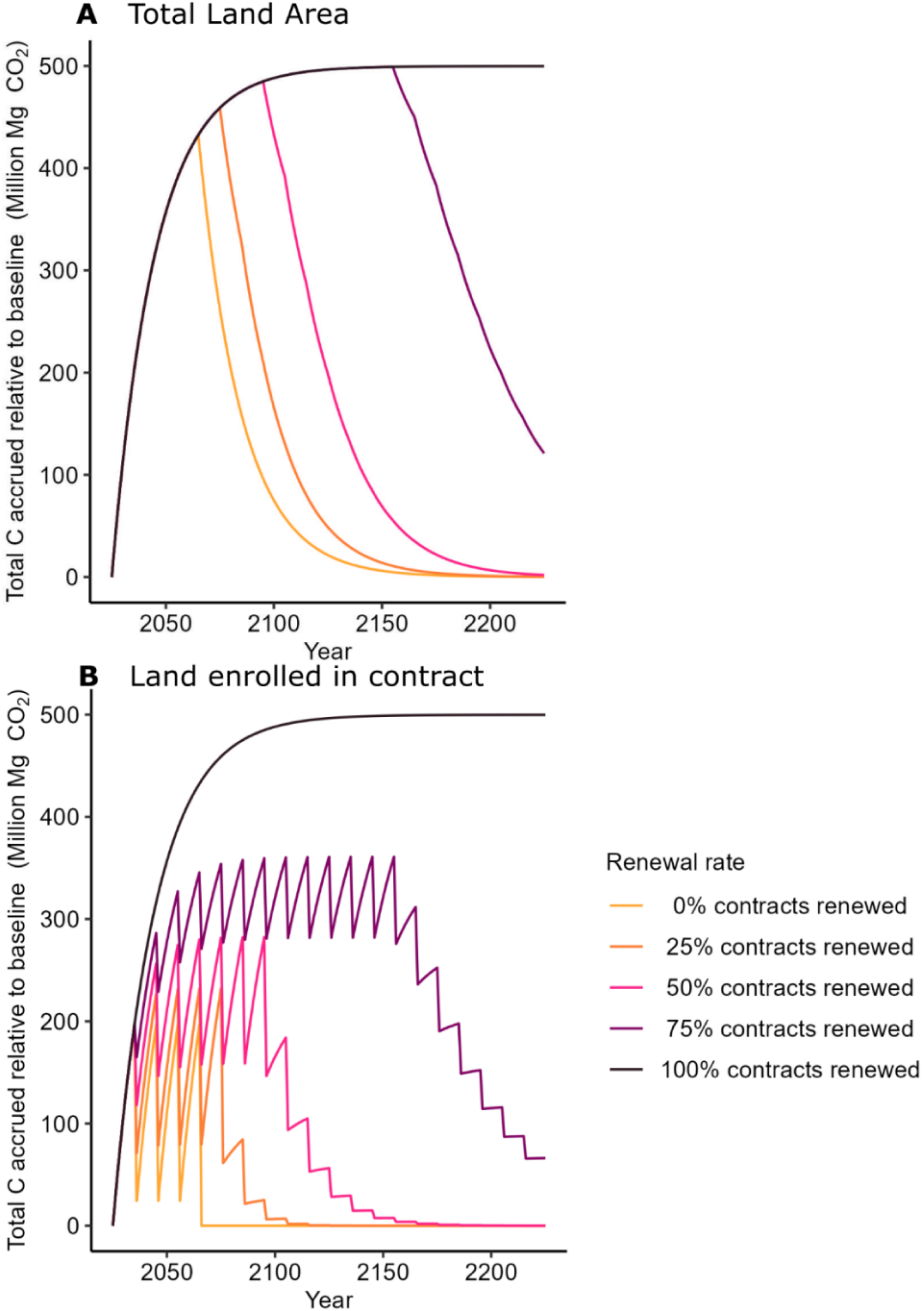
(A) Soil carbon balance relative to baseline across all land uses, assuming 0% contract renewal (yellow, i.e., all land under management for reversible CDR is reverted back to business as usual after one contract length), 25% (orange), 50% (pink), 75% (maroon) contract renewal rates (i.e., 75% of land enrolled in contracts for management for reversible CDR is renewed for at least one additional contract length), and 100% (black), where the amount of land that initially went under contract is continually renewed and remains under contract throughout the entire time period. (B) shows the total carbon balance solely for land enrolled in contracts under the same contract renewal scenarios as the top figure. Total overall C balance is highest for land with the highest renewal rates (e.g., 100%), due to the maintenance of previously accrued soil C. Oscillations in C stock occur at the end of each contract period, in this case every 10 years.

Atmospheric carbon dynamics changed substantially when durable carbon storage was available. We projected that the proportion of released soil carbon that would be removed via DACS grew as a logistic function, reaching 95% in either 2050 (early DACS transition) or 2075 (late DACS transition). All durable storage cases led to net carbon removal. However, the total amount of carbon removed depended on the maintenance of a contract (renewal rate and contract length) for reversible soil-based storage (Figure 2, Figure S2). Only in the case of low renewal rates (0%) or a short contract length of 5 years (see Supporting Information) did significant soil carbon re-emission occur faster than the growth of DAC capacity, reducing long-run CDR. When soil carbon contracts were maintained for longer than 5 years, the delayed release of accrued carbon allowed DACS capabilities to scale enough to instantaneously capture soil carbon emissions.

### Temperature Reductions

Temperature reductions were predictably larger and longer-lasting when reversible storage contracts were maintained longer periods. The maximum reduction in temperature achieved from continuously implementing the same projects over 22.75 million ha was 227 μ°C in 2500 for SSP1-2.6 (solid line in Figure 3), which is equivalent to approximately 1.4% of the average annual increase in global temperature since 1975.^45^ Allocation of all resources to rapid soil carbon accrual at the expense of maintaining stored carbon (i.e., 0% contract renewal rates) reduced climate mitigation impacts (>70 μ°C warmer in 2075). Without durable storage, all scenarios with renewal rates less than 100% resulted in only reversible warming reductions. The duration of storage was a strong control on warming reduction. For instance, in the scenario with 10-year contracts and 75% renewal rates, temperatures were reduced below the baseline well after 2150 (Figure 3). However, under the lowest renewal scenarios, most of the stored SOC was re-emitted prior to peak warming within 100 years, such that the cooling effect of soil carbon storage declined to zero by the end of the century.

**Figure 3:**
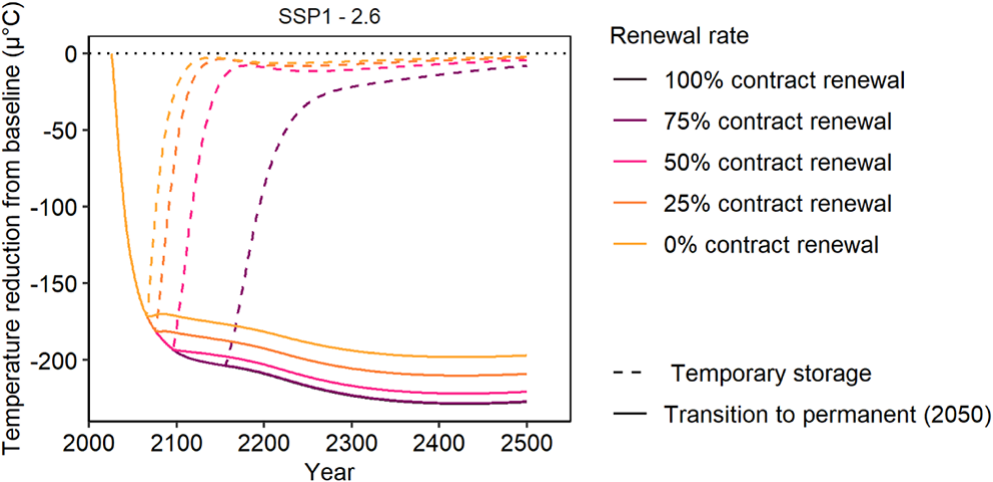
Warming reduction in μ°C compared to the baseline SSP1–2.6 for scenarios with increasing maintenance of 10 year contracts (darker-colored lines represent higher contract renewal rates). Dashed lines indicate temperature trajectories for reversible storage where contracts can expire. Solid lines represent temperature trajectories for continuously implemented projects (black),or for the full transition of released reversible carbon storage to durable storage (Early DACS Transition scenario). For the case of a 100% renewal rate, continuous reversible storage has the same temperature reduction trajectory as the durable storage trajectory for the same amount of carbon. Durable storage transition scenarios store released carbon in durable storage, with the magnitude of carbon storage depending on the maximum net carbon removed and stored in each contract renewal rate scenario.

Introducing durable storage via DACS significantly increased potential temperature reductions (Figure 3–solid lines). Greater temperature reductions occurred at higher renewal rates. With durable storage as a backstop, strategies that succeed in maintaining soil carbon storage (50–75% renewal rates) resulted in greater temperature reductions than strategies that did not maintain soil carbon storage, e.g., 227 μ°C (75% renewal rate) vs. 197 μ°C (0% renewal rate) in 2500 for Early DACS Transition (Figure 3). Lower renewal rates can be compensated for with longer contracts to maintain storage and achieve temperature reduction (Figure S6). These results indicate that even with the transition to durable storage, temperature reductions were sensitive to the successful maintenance of reversible soil carbon storage.

### Economic Costs

The cost of continuously “renting” reversible CDR via cover cropping is lower than an immediate transition to durable storage via DACS, but only under the assumption that soil carbon can be maintained for centuries. In the “rent-only” scenario, we implemented one carbon project on the 22.75 million ha and paid for maintenance indefinitely (100% contract renewal). In the “buy-only” scenario, we replicated the carbon trajectory by purchasing durable storage from the beginning, assuming the DACS industry can operate on that scale. In 2050, the net present value for rent-only storage was 0.17–0.70 billion USD per μ°C across CO_2_ price pathways, compared to 1.00–2.60 billion USD per μ°C for buy-only durable storage (Table 1). By 2500, the net present value of the rent-only storage scenario was 0.32–1.31 billion USD per μ°C, compared to 1.37–2.19 billion USD per μ°C for buy-only.

**Table 1:** Global Temperature Reduction Relative to a Baseline SSP1-2.6 Temperature Trajectory Given Durable Storage (Buy-Only) and 10 Year Reversible Storage Contracts (Rent-Only) from No Contract Renewals (0%) to Continuous Renewal Rates (100%)^a^

	**Buy-only**		**Rent-only**		**Rent-then-buy**	
Year	Warming reduced (μ°C)	Cost (Billion USD per μ°C)	Warming reduced (μ°C)	Cost (Billion USD per μ°C)	Warming reduced (μ°C)	Cost (Billion USD per μ°C)
	**0% Renewal**					
2050	141	1.84–1.01	141	0.19–0.75	141	0.21–0.93
2075	171	1.79–0.95	109	0.35–1.38	164–171	0.23–0.94
2100	171	1.79–0.95	22	1.74–6.96	163–171	0.23–0.94
2500	197	1.55–0.83	2	16.36–65.44	188–197	0.20–0.81
	**50% Renewal**					
2050	141	1.01–2.63	141	0.18–0.73	141	0.21–0.93
2075	180	0.94–2.65	180	0.24–0.95	180	0.22–0.93
2100	194	0.9–2.71	178	0.30–1.20	194	0.21–0.89
2500	221	0.83–2.42	5	12.33–49.35	221	0.19–0.78
	**100% Renewal**					
2050	141	1.00–2.60	141	0.17–0.70	–	–
2075	181	0.93–2.61	181	0.23–0.91	–	–
2100	195	0.92–2.67	195	0.27–1.07	–	–
2500	228	1.37–2.19	228	0.32–1.31	–	–

a The range of net present values for each year was divided by the warming reduction in that year to give the cost of cooling in billion USD per μ°C. The range of net present value for buy-only is based on the minimum and maximum values depending on three future DACS cost trajectories, and the rent-then-buy ranges span the same DACS cost trajectory ranges, three future CO_2_ price trajectories, as well as both the Early and Late DACS Transition pathways. All costs are in real 2025 dollars. Rent-then-buy outcomes are given for 0% and 50% reversible contract renewal rates. Rent-then-buy outcomes are equivalent to rent-only outcomes for 100% renewal, as all carbon stays in reversible storage without any replacement of contracts for the 100% renewal rate.

When we allowed reversible CDR to expire over time without replacement by DACS, the economic cost of warming reduction increased substantially over the time considered (pink points in Figure 4). At low renewal rates (i.e., 0%–50% renewal), costs become relatively high by 2100 because the cooling effect of reversible carbon storage approaches zero. Low renewal rates cost 0.30–6.96 billion USD per μ°C reduction across CO_2_ price pathways in 2100 compared to lower costs of 0.23–1.07 billion USD per μ°C for higher renewal rates of 75–100%. This pattern was accentuated when we considered longer time frames due to both the cost of maintenance and diminishing temperature reductions as carbon is released. Although the relative ranking of cost per warming reduction remained the same across discount rates, the net present value was sensitive to the choice of discount rate, with higher discount rates leading to a lower net present value of CDR in the future (see Figure 5 for a comparison of cumulative cost per degree of warming reduction in 2100 across discount rates).

**Figure 4:**
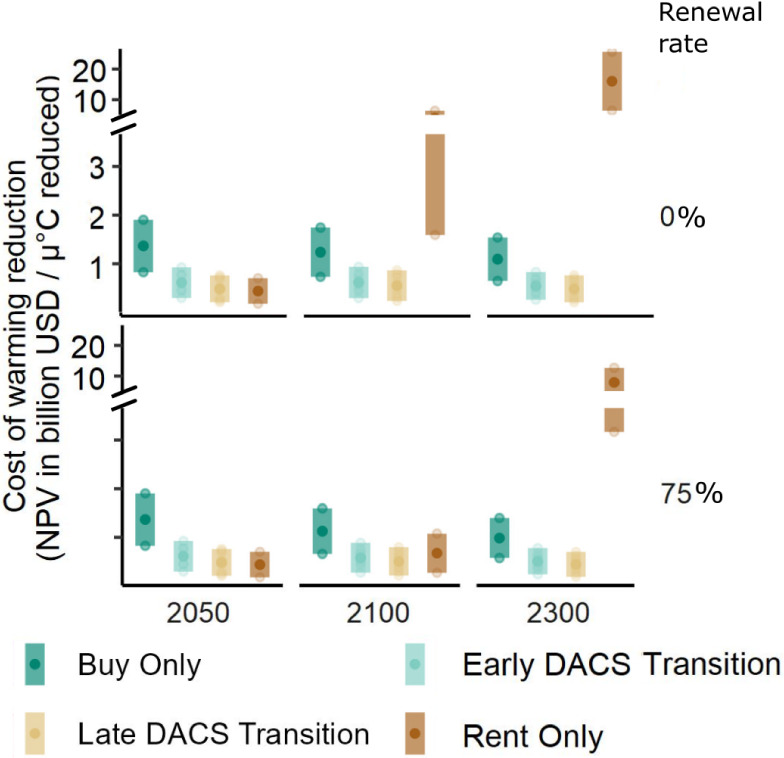
The cumulative cost of cooling is given as net present value per degree of warming reduced from an SSP1–2.6 baseline for each CDR scenario. Points are presented for 3 years on the *x*-axis. Note that net present value is calculated on a cumulative basis and thus is not an annual cost rate. Each panel represents a different contract renewal rate for reversible storage, increasing from top (0% renewal) to bottom (75% renewal). Reversible storage (“rent only”) without durable storage is represented as dark brown rectangles, which vary by projected future values of CO_2_ (low, medium, and high) and are independent of DACS cost uncertainty. The broken *y*-axis, with a zoomed-out scale at high costs, illustrates the trend over time of the cost of renting reversible storage, which increases as carbon is released and the temperature effect approaches zero and far exceeds the small-scale *y*-axis. Ranges for the durable storage scenarios, including the transition scenarios from reversible to durable storage by 2050 (Early DACS Transition; light teal) and 2075 (Late DACS Transition; light brown), and for the “buy-only” durable storage scenario (dark teal), are given as vertical rectangles, which represent the maximum and minimum calculated net present values given three trajectories for future CO_2_ prices and for future DACS costs (low, moderate, and high). Comprehensive graphics of all scenarios are in the Supporting Information (Figures S1-S6).

**Figure 5:**
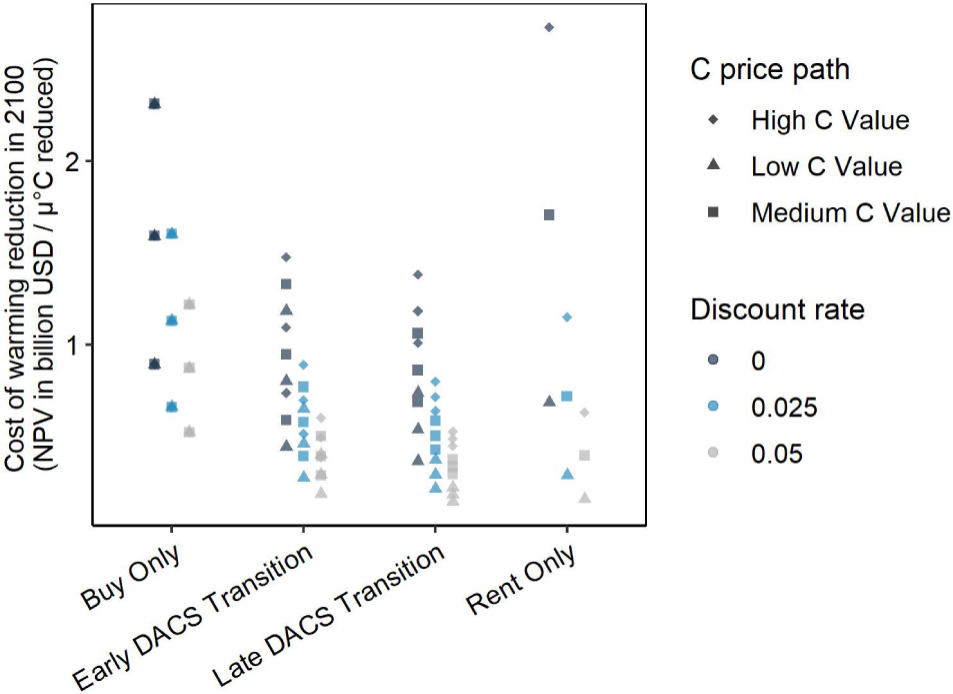
A comparison of the effect of discount rate (0%: dark blue; 2.5%: light blue; 5%: gray) on the cumulative cost of cooling is given as net present value per degree of warming reduced from an SSP1–2.6 baseline in 2100 for a 50% renewal rate scenario. Points are presented for four reversible-to-durable storage transition pathways on the *x*-axis, and points of the same discount rate vary across projected future CO_2_ values (low: triangles, medium: squares, high: diamonds). Points are overlapping in the “Buy-Only” and “Rent-Only” scenarios. A graphic of all scenarios and discount rates is in the Supporting Information (Figure S4).

Transitioning fully from reversible storage to durable storage within the century yields the most cost-effective storage pathways. For the Early DACS Transition, a contract length of 10 years with a 75% renewal rate led to a 2100 warming reduction of 227.5 μ°C at a cost of 0.49 billion USD per μ°C, whereas a 0% renewal rate led to a lower warming reduction of 197.1 μ°C and cost 0.53 billion USD per μ°C for the moderate DACS cost trajectory. Both rent-then-buy transition pathways with early and late logistic growth of DACS achieved the same temperature reductions as the buy-only durable storage pathway (227.2 μ°C for a 75% renewal rate), but for lower costs of 0.18–0.76 billion USD per μ°C compared to 0.56–1.37 billion USD per μ°C for durable storage only. As in the previous case with reversible storage only, cases with longer enrollment periods (i.e., higher renewal rates) were cheaper per μ°C and achieved a larger cooling effect, as reversible storage had to be replaced with durable storage earlier at a higher cost. With the transition to durable storage via DACS, the short-run outcomes by 2050 had the same temperature reduction of 141.4 μ°C and ranged in cost from 0.21–0.92 billion USD per μ°C across DACS costs, with identical carbon removal and costs across renewal rate cases. In these cases, costs increased as durable storage occurred through 2100–2200 and then decreased in the very long run with increased warming reductions.

## Discussion

Our results illustrate how a patchwork of reversible CDR projects can yield warming reductions over multidecadal time scales, but only if the patchwork is well-maintained. Consistent with earlier analyses,^11^ we found that even though carbon stored in agricultural soil is vulnerable to re-emission, lost carbon can be replaced by the expansion of CDR to different lands. The durability of ecosystem-based CDR is thus not only a function of biophysical factors but also depends on the rate at which CDR projects are replaced. Determining the value of ecosystem-based CDR in a net-zero world consequently requires accurate forecasts of how successfully C storage will be administered over the long term.

Our analysis also shows that transitioning from reversible CDR to durable CDR is less expensive than the extreme “rent-only” and “buy-only” strategies. The scenarios with a backstop for durable storage via DACS are like those proposed by Herzog et al. (2003), and illustrate the idea that reversible storage can act as a bridge to durable storage in the long term.^10,46^ While we focused on carbon sequestration in agricultural soils, other reversible CDR pathways, such as reforestation, may also serve as a bridge to durable CDR. The distinct contract structures and biogeochemical parameters associated with these pathways would lead to different timing and magnitudes of tradeoffs.

Our model of soil-based CDR potential over time depends on several assumptions regarding land manager behavior. For instance, we conservatively assumed that managers will only adopt a CDR practice if they have an economic incentive and that they immediately end the practice after the incentive ends. The first assumption is supported by studies showing that farmers are most likely to adopt a new practice when they will receive short-term economic benefits.^47,48^ The second assumption may be overly conservative: for instance, federal programs in the United States, such as the Conservation Reserve Program, have reported 20–60% of farmers maintaining practices after paid contracts expire.^49,50^ On the other hand, voluntary conservation practices are much more sensitive to reversal during years with productive weather conditions and profitable crop prices.^49^ This suggests that contracts offer some protection against short term management reversals, and that it would be risky to assume the continuation of carbon storing practices after payments have ceased.

Critically, we also assumed that land cannot be re-enrolled in a CDR program following the re-emission of stored carbon. This assumption has a major impact on our analysis because it ultimately forces re-emission to occur. We imposed this limit to illustrate the effect of the correlated risk across the CDR patchwork. Our analysis supports the idea that temporary cooling from reversible CDR will provide lower value over the long term than strategies that incorporate durable CDR; however, this discrepancy might be even more severe in reality. A rebound in warming following re-emission of CO_2_ stored in ecosystems might occur in a more vulnerable future world with increased population, income, and exposed assets. This scenario would lead to higher social costs of future climate damages. While our modeling does not explicitly incorporate these social cost dynamics, it is important to recognize that the welfare implications of reversible versus durable carbon storage depend not only on physical temperature outcomes but also on the timing of the associated damages. Our findings are decoupled from political and ecosystem constraints, as they are a controlled analysis of the sensitivity of macroeconomic frameworks on future costs and temperature. Incorporating this framework into an integrated assessment model (IAM) would advantageously allow us to explore the interactions between demand, necessity, and scalability of both reversible and durable CDR and storage over time and understand the extent to which ecosystem storage could effectively act as a bridge solution. We hope that these findings may ultimately improve the simulation of ecosystem-based reversible storage within IAMs in the future.^7,27^

Relying on reversible storage as a bridge to durable storage appears to be cost-effective in our analysis, but this result only emerges under the assumption that both commitments to the maintenance of reversible storage and the transition to durable storage are honored. One strategy to manage the risks associated with phased commitments, which include an initial period of ecosystem carbon rental, would be to apply the principle of “like-for-like” carbon accounting schemes, which balance ecosystem CDR against reversible emissions from land use.^7,18–20^ In this case, the total amount of rented carbon storage would be limited to neutralizing land-use emissions at a jurisdictional level, and a lapsed commitment would not be responsible for unmitigated fossil fuel emissions. Another approach to minimizing risk would be to limit the duration of reversible storage by replacing ecosystem C with geological storage faster—but this option would come at a higher cost (Figure 4). Combining near-term ecosystem storage with a durable storage backstop thus represents a spectrum of strategies, with a tradeoff between high-cost, low-risk early adoption of geologic storage and lower-cost, higher-risk delayed adoption.

We made some modeling assumptions that are optimistic and should be considered carefully before applying lessons from our analysis to the real world. First, the cost declines that we assumed for durable storage via DACS technologies rely in part on the scaling up of the deployment of durable storage, even though we treat these costs as exogenous for the purposes of this analysis. There is some risk that delaying the adoption of durable storage using reversible removals, in turn, delays the deployment of and associated cost reductions in durable storage. To avoid this, there is a role for institutions to invest some resources in developing durable storage technologies today, even if these may not represent the most cost-optimal near-term mitigation approach compared with reversible removals. Second, we assume immediate, nondelayed CDR action within a net-zero carbon budget, emphasizing comparison of the blend of reversible vs durable storage rather than the amount and timing of storage. We acknowledge that relaxing the assumption of a fixed carbon budget would result in different cost and climate implications (see Matthews et al., 2022 ^6^) but do not consider delayed action, as this would require an additional analysis of the social and financial costs of delaying warming reductions.

Finally, the value of any form of storage—whether reversible or durable—is strongly dependent on the underlying mitigation pathway.^51^ Removals are less effective in reducing global temperatures under high-emissions pathways, and both horizontal stacking of reversible removals and durable removals are more expensive than many other forms of mitigation in the near term. Policymakers need to balance both responsibility and cost when determining the optimal levels of CDR deployment, and the value of CDR will tend to increase as the world gets closer to net zero^52,53^ and other lower-cost mitigation options are exhausted, both at the sector and economy-wide levels.

## Implications

Stacking reversible soil CO_2_ removal and storage over time can contribute to reducing peak temperatures and maintaining carbon storage for longer, leading to larger and more sustained temperature reductions. Renting reversible CO_2_ removal can be a bridge to buying durable CO_2_ storage in the long term, yielding temperature reductions at a lower cost than alternative storage pathways. However, this strategy requires that the commitment to transition from reversible storage to durable storage be upheld. Consequently, this strategy is riskier than the immediate deployment of durable storage because maintenance of reversible storage and commitments to durable storage might lapse. This risk would likely shorten the optimal deployment timelines for reversible storage. Quantifying this risk accurately is likely essential for using ecosystem CDR as an effective bridge to durable carbon storage.

## Supplementary Material





## Data Availability

Model code and associated data will be made available on Dryad at the time of publication.

## Notes

Model Code: containing R code (ZIP)
Model parameterization and detailed descriptions, cost trajectories, comprehensive scenario results, decarbonization baseline, and contract length analyses (PDF)
