# Supplementary material for: The Value of Reversible Carbon Storage in a Zero-Emissions World
Source: Environ Sci Technol. 2026 Jun 7;60(24):17281–90. doi: 10.1021/acs.est.6c00333 (PMC13296497; doi:10.1021/acs.est.6c00333)
Supplement: Supplementary file 2 [file es6c00333_si_002.pdf]

Supplemental Information for *The value of reversible carbon storage in a zero-emissions world*

2026

Authors: Allegra Mayer[1], Jerome Dumortier[2], Zeke Hausfather[3], Jennifer Pett-Ridge[1,4,5], Eric Slessarev[6,7]

Affiliations:

[1] Lawrence Livermore National Laboratory, Physical Life Sciences Division, Livermore, CA, 94551, USA

[2] Indiana University Indianapolis, O'Neill School of Public and Environmental Affairs, Indianapolis, IN, 47405, USA

[3] Stripe Inc., South San Francisco, CA, 94080, USA

[4] Berkeley Earth, Berkeley, CA, 94705, USA

[5] Life and Environmental Sciences Department, University of California, Merced, CA, 95343, USA

[6] Innovative Genomics Institute, University of California, Berkeley, CA, 94704, USA

[7] Yale University, Department of Ecology and Evolutionary Biology, New Haven, CT, 06511, USA

[8] Yale University, Yale Center for Natural Carbon Capture, New Haven, CT, 06511, USA

*Corresponding author: mayer25@llnl.gov

Supplementary Information (pages S1-S10)
Text 1-7 (this pdf file)
Figure S1-S6 (this pdf file)
Code in separate zip file
Readme.txt

1. Run SOC and patchwork models.R
2. FaIR patchwork analysis.py
3. Post-processing of Fair Model.R
4. Cost of cooling economic analysis.R
5. Cost of cooling economic analysis with comparison of results vs practice.R

1 Soil organic carbon model parameterization

We simulated soil carbon accrual and release with implementing cover crop management using a linear one-pool model. To keep this analysis consistent with notation typically used in ecosystem science, all pools, rates, and parameters in this analysis are in units of carbon mass (C), not CO_2 equivalent mass (CO_2-e); units were then converted to CO_2-e before being passed to the economic model. C stocks were governed by equation (2) from the main text

$$C_{soil}(t) = \frac{n}{k}I - \left(\frac{n}{k}I - C_0\right)e^{-k(t-t_0)} \quad (2)$$

Where C_0 is the SOC stock (Mg C ha^{-1}) at time t_0) n is the fraction of carbon input that enters the soil (unitless), and k is a first order decay constant (y^{-1}). We parametrized this equation so that the steady state SOC stock without cover cropping (defined as C_{bau}) would be 53 Mg C ha^{-1}, which corresponds to the mean organic carbon stock in the top 20 cm of cropland soil in US croplands [1]. The decay constant k was parameterized as 0.05 y^{-1}, based on published values for grass planted on cropland soil [2,3]. The I and n parameters were constrained by the fact that at steady state C_{soil} equals I*n/k. Assuming a value of 0.5 for n and considering our assumption that C_{bau} = 53 Mg C ha^{-1} at steady state, I in the business-as-usual scenario (I_{bau}) equaled 5.3 Mg C ha^{-1} y^{-1}. This value is on the high end annual carbon inputs in midwestern corn-soybean systems [4]. We increased the value of I by 0.6 Mg C ha^{-1} yr^{-1} under cover cropping so that the change in carbon stocks would be approximately 0.3 Mg C ha^{-1} y^{-1} for the first five years after initiating the practice (I_{cc} = 5.9 Mg C ha^{-1} y^{-1}). An increase in SOC of 0.3 Mg C ha^{-1} y^{-1} over this timeframe is consistent with mean estimates of soil carbon accrual from implementing cover crops on annual croplands, and may be increased or decreased depending on species and timing of cover crop[5]. We note that the 0.6 Mg C ha^{-1} y^{-1} increase in inputs with cover crops is comparable to the mean root carbon inputs from cover crops in the United States [6], but is less than the expected mean biomass inputs when accounting for both above- and below-ground cover crop biomass (~ 2 Mg C ha^{-1} yr^{-1}) [6,7]. This model accounts for the variable rate of soil carbon accrual over time, where a patch that has been contracted three times in a row accrues at a slower rate than a patch of land contracted for the first time.

The SOC stored per unit area over time (C_{soil}, Mg ha^{-1}) was obtained by constructing and parametrizing a piece-wise function from Equation 2:

$$C_{soil} = \begin{cases} \frac{n}{k}I_{bau} = C_{bau} & if\ t \leq t_e \\ \frac{n}{k}I_{cc} - \left(\frac{n}{k}I_{cc} - \frac{n}{k}I_{bau}\right)e^{-k(t-t_e)} & if\ t_e < t \leq t_u \\ \frac{n}{k}I_{bau} - \left(\frac{n}{k}I_{bau} - C_u\right)e^{-k(t-t_e)} & if\ t > t_u \end{cases} \quad (3)$$

Where t_e is the time when the patch enrolls in cover cropping and t_u is the time when it unenrolls. The quantity C_u was defined as the SOC stock when the patch reverts from cover cropping to business-as-usual management and was obtained by solving Equation 2 at time = t_u:

$$C_u = \frac{n}{k}I_{cc} - \left(\frac{n}{k}I_{cc} - \frac{n}{k}I_{bau}\right)e^{-k(t_u-t_e)} \quad (4)$$

Net SOC storage (C_{store}, Mg ha^{-1}) was then calculated by subtracting C_{bau} from the solution to Equation 3.

2 Patchwork model description

The patchwork land-use model tracks area of land under each management class (business as usual, enrolled in cover cropping contract, and land with lapsed cover cropping contract), and tracks carbon stocks in each of those land classes over time. For example, for the contract length of ten years and the renewal rate of 25%, 22.75 million hectares were put under new cover cropping contracts in 2025, whereas 17.1 million hectares were put under new contracts and 5.7 hectares were renewed in the years 2030, 2035, 2040, 2045. The land area in each patch (A_p) is time invariant because each patch is defined in terms of a unique combination of time of enrollment (t_e) and time of unenrollment (t_u). in addition to the renewal rate (r) and the contract length (l):

$$A_p = \begin{cases} A_t(1-r)r^{m-1} & if\ t_e = 0 \\ A_t(1-r)^2 r^{m-1} & if\ t_e > 0 \end{cases} \quad (5)$$

Where the variable *m* is the number of contract periods that had elapsed when the patch was unenrolled:

$$m = \frac{(t_u - t_e)}{l} \quad (6)$$

We calculated the total amount of C stored in the patch over time ($C_{patch,}$ Mg C) by multiplying A_p by C_{store}. The patchwork was defined for all combinations of t_e and t_u constrained by the contract length, with the constraint that t_e could not be greater than the simulation year in which all available land had been under contract once. We summed C_{patch} across all patches to obtain total CDR over time.

3 Transition from Reversible to Durable CDR

There is a significant uncertainty about the diffusion path of DACS over time. To cover a range of possibilities and also the two extreme cases of 100% diffusion in 2025 and no diffusion at all over
the time period until 2500, we assumed various diffusion pathways. In the *Rent Only* scenario, we assumed that carbon is sequestered in soils until 2500 with no DACS available. The scenario *Buy Only* represents the opposite case where all carbon removed from the atmosphere is durably placed in geologic storage. The logistic growth scenarios assume a S-shaped growth until 2050 or 2075 (Early and Late DACS Transitions) in the percentage of carbon that is removed through DACS (**Figure S1a**). Those growth functions are consistent with technology diffusion curves (Rogers, 1962). The logistic growth curves are written as follows:

$$s_t = \left[1 + \left(\frac{1 - s_0}{s_0}\right) e^{-rt}\right]^{-1}$$

with *r* being calibrated such that $s_0 = 0{\cdot}01$ and $s_t = 0{\cdot}95$ in 2050 or 2075.

The results of the various DACS diffusion pathways depended on both the backstop for durable

geologic storage (2025, 2050, or 2075), and the renewal rate for reversible carbon storage, as this determined the timing of carbon released from reversible storage and replaced with durable storage (**Figure S2**).

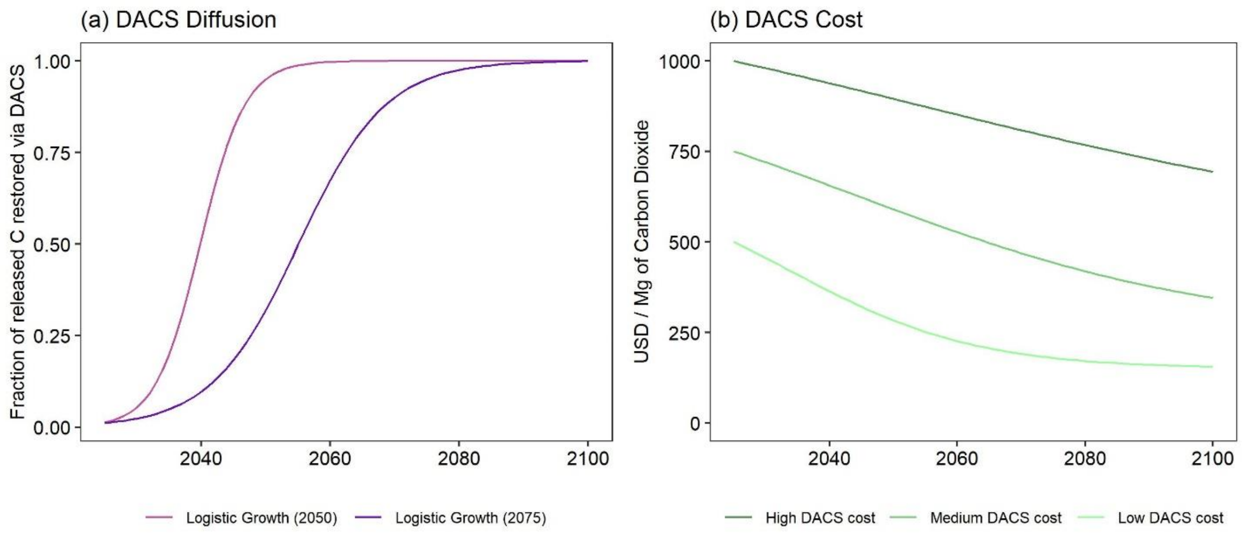

Figure S1. DACS diffusion trajectories representing the use of durable carbon removal as opposed to reversible removal. The logistic growth curves are based on Equation 3. Except for the rent only scenario, 100% DAC use is assumed past 2100.

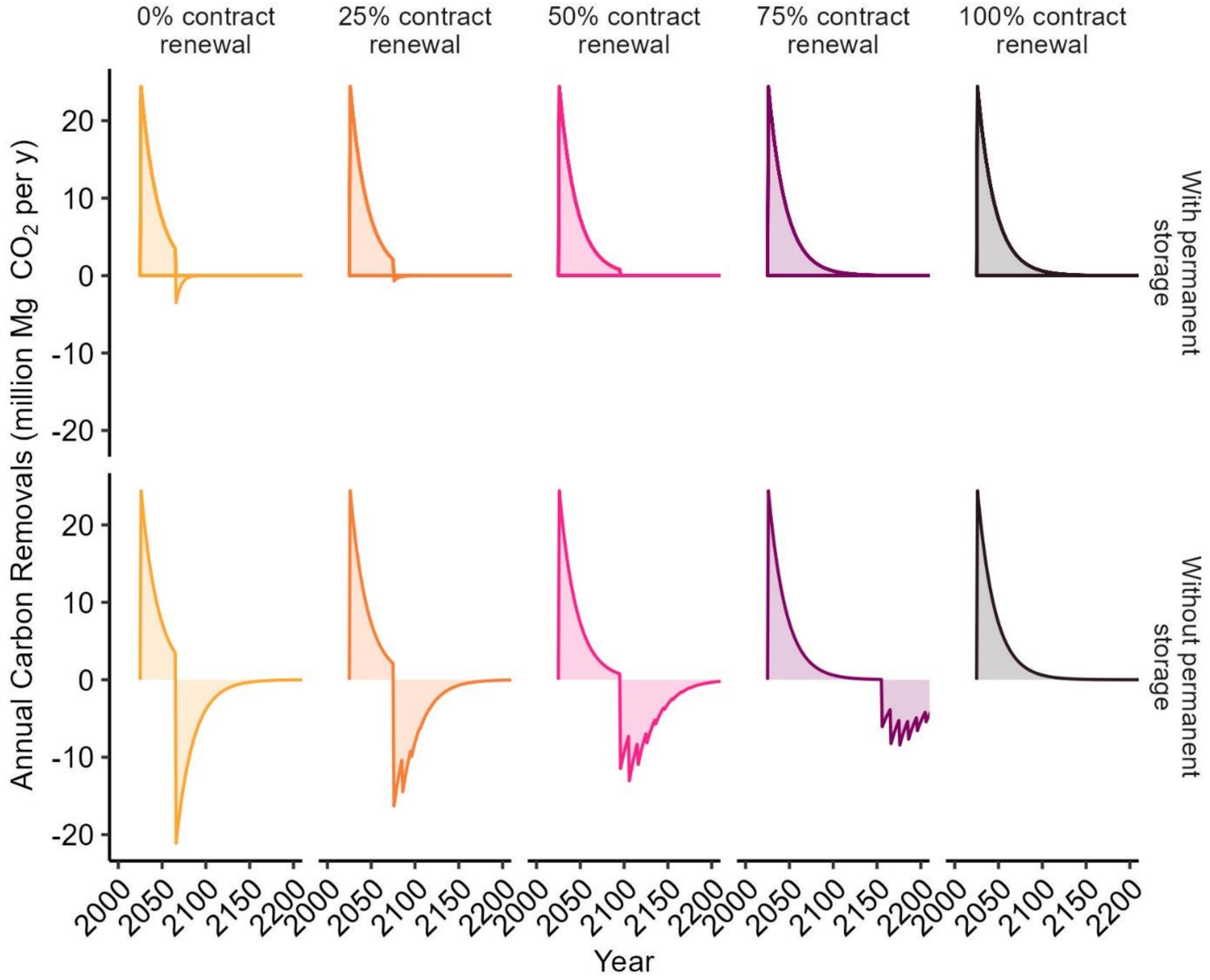

Figure S2: The rate of annual atmospheric carbon removal is shown here for contract cases (0% - 100% renewal rates from left to right) for each scenario of growth of durable storage via DACS. In the bottom panels, lines show the "rent only" cases of reversible carbon removals without durable storage, leading to remission (negative carbon removals) in the second half of the century, except in the 100% renewal case. In the top panels, the solid lines represent "buy only" where all carbon that would have been removed temporarily in soils is instead removed via DACS from the beginning, and "rent-then-buy" bridge scenario where released reversible carbon is replaced with durable storage by 2050 or 2075.

4 Direct Air Capture Cost Trajectories

The technology considered for durable CO_2 removal from the atmosphere is Direct Air Capture with subsequent geological storage (DACS). There is significant uncertainty regarding the level, timing, and path of achieving long-run cost. Current cost estimates are in the \$600–\$1000 range per metric ton (Mg) of CO_2-e removed.[8*] The cost range in the long-run varies widely from below

$60 by 2040 to $280 by 2050 per Mg of CO_2-e.[9,10] We generate three potential cost paths with starting values for the low, medium, and high DACS cost curves of $500, $750, and $1,000 per Mg^{-1} CO_2-e, respectively. The long-run prices are
$150, $250, $500 per Mg^{-1} CO_2-e (Figure **S1b**). The cost trajectories are modeled as follows:

$$C(t) = L + (U - L)(1 + \exp\left(\frac{ln(2)}{k}(t - k)\right))^{-1}$$

where the values of U, L, and k are represented in the table below. The above values, correspond to starting cost of $500, $750, and $1000 Mg^{-1} CO_2 in 2025 and long-run cost of $150, $250, and $500 Mg^{-1} CO_2 in 2300 for the low, medium, and high DAC cost scenario, respectively. The corresponding cost path is shown in Panel (b), Figure S1.

Scenario	U	L	k
Low DAC Cost	688	150	10
Medium DAC Cost	1009	250	20
High DAC Cost	1256	500	30

[*] See also Unlocking the potential of direct air capture: Is scaling up through carbon markets possible? published by the International Energy Agency on 11 May 2023.

5 Carbon Price Path and Payment Policy

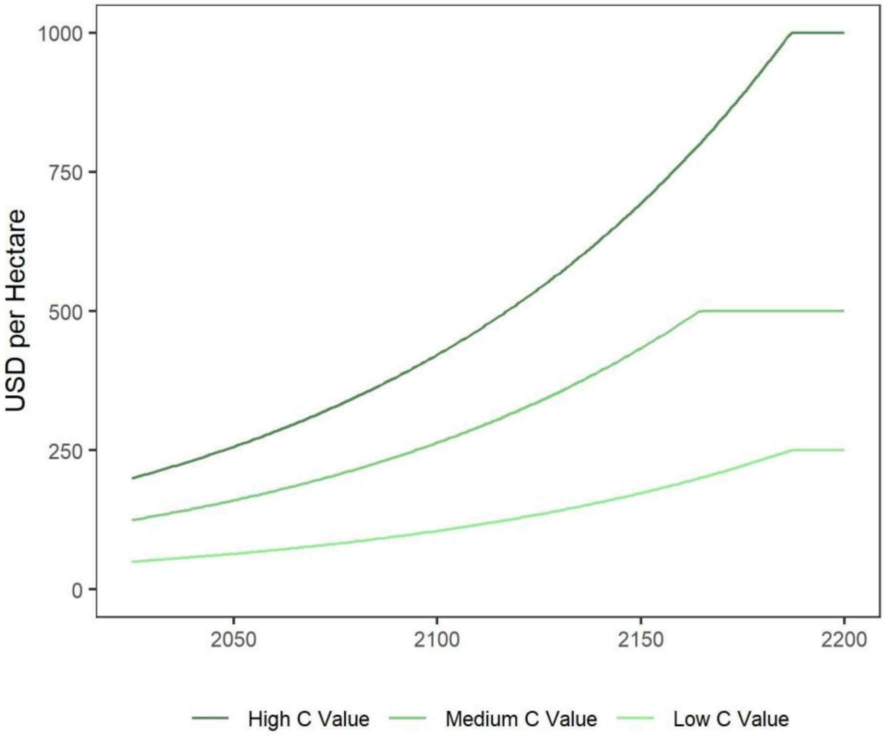

Figure S3. Future trajectories for the value of implementing a practice to remove and store CO_2 in a hectare of land depending on the future value of CO_2 (carbon price paths), given in $ per hectare.

We considered three carbon value paths, labelled *Low CO_2 Value*, *Medium CO_2 Value*, and *High CO_2 Value*. The carbon price is in USD per Hectare. The growth rate for each path is set to 1% and the starting values are $50, $125, and $200 per Mg CO_2-e for low, medium, and high price paths, respectively. For the three price paths, we assume that the maximum CO_2 value is $250, $500, and $1000 per Mg CO_2-e for the low, medium, and high CO_2 value respectively. As with other components of our analysis, there is substantial uncertainty regarding the payment to farmers for reversible carbon removal and storage. Note that unlike in Herzog et al. (2003), there is the possibility that the carbon price grows at a different rate than the discount rate. The discount rates increase from 0% to 5% in steps of 2.5 percentage points. The resulting carbon price trajectories are depicted in **Figure S3** and are in line with the values presented in Strefler et al. (2021). Though we present results for only one discount rate (2.5%), we also analyzed the net present value of each scenario under five different assumed discount rates (**Figure S4**).

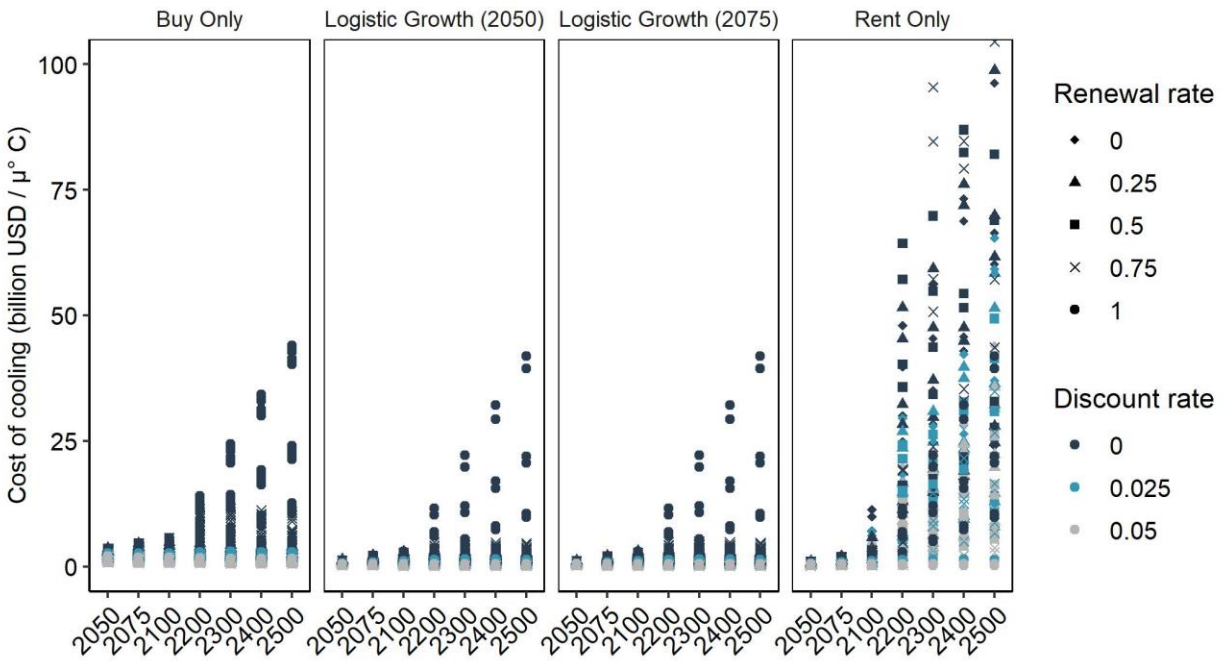

Figure S4: Net present cost of implementing a performance-based practice (y-axis) vs. the resulting temperature reduction from baseline (x-axis) was similarly sensitive to the rent vs. buy transition trajectories across all discount rates analyzed, including 0 (darkest blue), 0.025 (teal), and 0.05 (grey).

Regarding the payment policy, we differentiate between two policy cases: *Results-Based Payments* (not presented here) and *Practice-Based Payments*. In the case of *Practice-Based Payments*, the landowner receives a payment per hectare to implement a certain practice. Although those payments are fixed over time, land managers receive a per hectare payment even if the soil is no longer accruing carbon. This policy avoids the reversal of the carbon-removal practice once the soil has reached its steady state in terms of carbon stock. The three per-hectare payment amounts are assumed to be $50, $125, and $200. In the case of *Results-Based Payments*, payments to the landowner are based on the amount of CO_2 sequestered. In any case, the landowner is compensated for carbon accrued and does not need to pay in the case of carbon release. The result-based CO_2e also assume that carbon released gets immediately stored in geologic carbon. That is, all the negative flows from land are stored via DACS.

Results-based payments require accurate measurements of CO_2 stored in soils or forests, which can be expensive and time-intensive with current measurement techniques. In addition, a project manager's incentive to continue a practice may decrease over time, since the carbon removal rate and the associated carbon payments would decrease as soils approach equilibrium [11]. In contrast to results-based payments, practice-based payments can potentially delay the re-emission of carbon since the incentive payment to the land manager continues over time, and does not approach zero in the long term. While practice-based payments are likely to encourage a practice

for a longer duration, for the same reason they are less efficient at removing CO_2 per dollar invested than payments for performance [12].

Paying for results of CDR rather than paying for the practice change over an area increases the efficiency of reversible storage in a world where all patches can remove C at the same rate Under practice-based payments, a standard mean value would be paid on a per-hectare basis across a given region and for the entire time of contract. At large area scales, payments for the mean value of CDR may reasonably capture the total CDR value across the heterogeneous landscape of agricultural soil. However, the representativeness of those per-area payments over time will change dramatically as the soil carbon trajectory transitions from the initial accrual phase to the maintenance phase as soils approach equilibrium. As the constant practice-based payments begin to pay more for maintenance than annual removals (as per results-based payments), especially in the case of higher renewal rates, the results-based payments would approach zero, making them more cost-efficient if the practice can be maintained.
While cost efficiency is higher under results-based payment schema, accurate measurement and verification tools present challenges to actually implementing such a practice at large scales. Also, results-based payment for reversible storage could potentially induce moral hazards of incentivizing rapid gain and loss of soil C, rather than incentivizing maintenance of accrued soil C once accrual rates approaches an equilibrium.

6 Decarbonization baselines

We present in the main text values for a low-emissions world as projected by Shared Socioeconomic Pathway 1 (SSP1-2.6), but also analyzed results for a moderate emissions world (SSP2-4.5). The temperature response of each rent-to-buy transition pathways is given in **Figure S5** for temperature baselines from both a moderate future shared socioeconomic pathway (SSP2 - 4.5) and a strong decarbonization pathway (SSP1 - 2.6). In every case, warming reductions from baseline are greater under SSP1 - 2.6, emphasizing the importance of combining *both* decarbonization and CDR strategies.

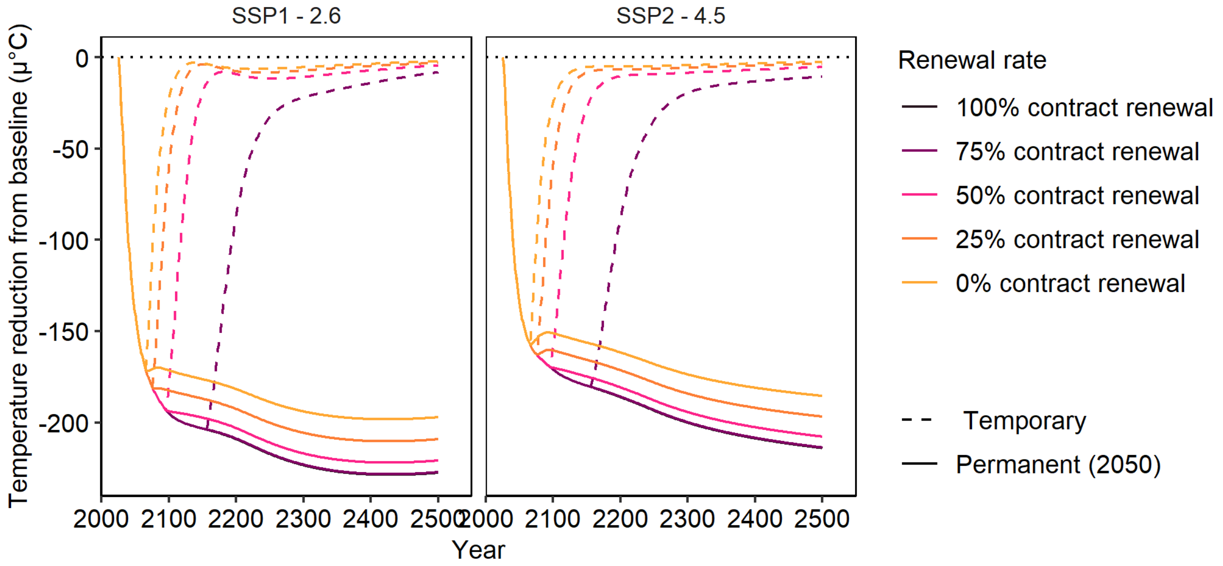

Figure S5: Comparison of warming reduction relative to baseline (SSP 1 - 2.6 , left and SSP2 - 4.5, right; y-axis) over time (x-axis) for 10 year contracts. The reversible "rent only" scenario is shown in dashed lines, with the durable storage "buy only" given in solid lines. Five contract maintenance experiments, each representing a different contract renewal and thus initial CO_2 removal rate, are represented for the rent or buy scenario. The experiments represent a range of removal rates versus storage maintenance: high initial removal rates and no maintenance for 0% contract renewals (yellow), 25% contract renewals (orange), 50% contract renewals (pink), 75% contract renewals (magenta), and maintenance of storage only with no shifting contracts in the 100% contract renewal experiment (black).

7 Contract lengths

Maintenance of reversible carbon storage through cropland management can be simulated by extending contracts two ways: renewal rate of finite contracts or the duration of the contract. Because both mechanisms can increase or decrease storage maintenance, we chose renewal rates as a primary example to demonstrate a range of storage durations. Increasing contract lengths has the same principle effect on carbon accrual in patches, temperature impacts, and net present values. **Figure S6** shows the factorial effect of renewal rates and contract lengths on temperature reduction from both baseline emissions pathways. We avoided making specific assumptions regarding the duration of soil-based CDR contracts or the rate at which contracts are renewed, instead exploring a range of contract lengths and renewal rates. While we explored contract lengths up to 20 years, shorter contracts are economically preferred for land managers under results-based payment frameworks due to declining soil carbon accrual rates and rising marginal costs.[11] This is consistent with the observation that shorter contracts are typical of the voluntary carbon market.[13] Even under payment-for-practice framework, long contracts that ensure maintenance of carbon storage are not commonly tenable in private agricultural settings. Shorter contracts (5-10 years) may thus be more representative of reality. On the other hand, intermediate to high renewal rates (50-75%) may not be unrealistic given that a majority of landowners prefer to re-enroll in practice-based conservation programs over time.[14,15]

Nonetheless, relying on the same patch of agricultural land to be continuously managed for soil-based CDR indefinitely is unrealistic due to landowner preferences for short contracts[16] and the challenge of predicting future landowner behavior. Shifting patchworks of management, corresponding to renewal rates less than 100% in our analysis, are more realistic.

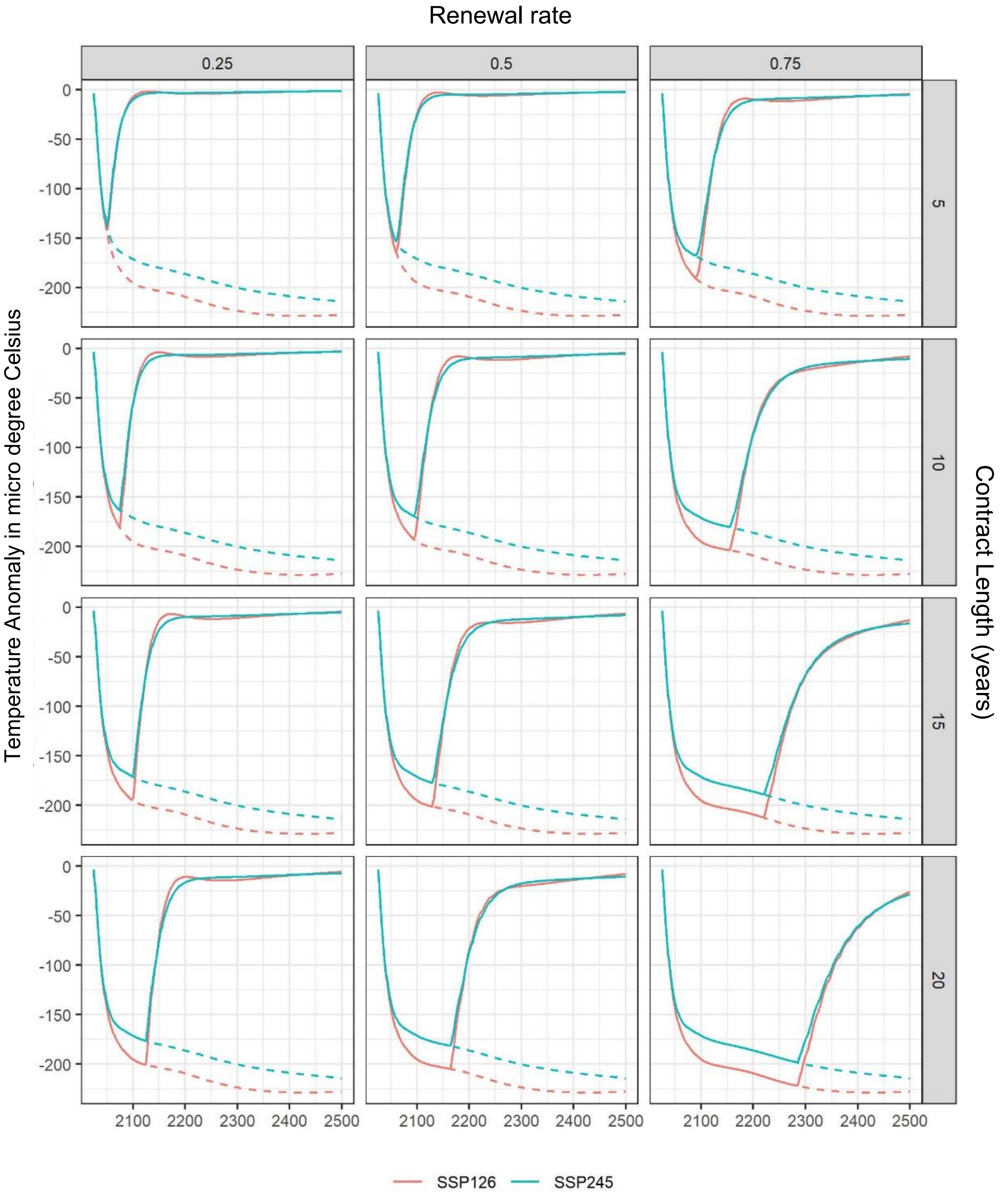

Figure S6: Temperature reductions from baseline scenarios (SSP1 -2.6, blue and SSP2 -4.5, red) depend on how long carbon can be maintained in reversible storage, for "rent only" cases, when no durable storage is available. Reversible storage can be maintained through increasing renewal rates, given as fractions for each column increasing from 25% renewal on the left to 75% renewal on the right, or through increasing contract lengths. Contract lengths increase from 5 years in the top row to 20 years in the bottom row. Dashed lines indicate 100% renewal rate for

continuously implemented projects, while solid lines indicate the temperature reduction for the combination of renewal rate and contract length.
